# An efficient viral vector for functional genomic studies of *Prunus* fruit trees and its induced resistance to *Plum pox virus* via silencing of a host factor gene

**DOI:** 10.1111/pbi.12629

**Published:** 2016-09-29

**Authors:** Hongguang Cui, Aiming Wang

**Affiliations:** ^1^ London Research and Development CentreAgriculture and Agri‐Food Canada (AAFC)LondonONCanada

**Keywords:** functional genomics, *Prunus necrotic ringspot virus*, *eukaryotic translation initiation factor 4E*, virus‐induced gene silencing, viral vector, sharka

## Abstract

RNA silencing is a powerful technology for molecular characterization of gene functions in plants. A commonly used approach to the induction of RNA silencing is through genetic transformation. A potent alternative is to use a modified viral vector for virus‐induced gene silencing (VIGS) to degrade RNA molecules sharing similar nucleotide sequence. Unfortunately, genomic studies in many allogamous woody perennials such as peach are severely hindered because they have a long juvenile period and are recalcitrant to genetic transformation. Here, we report the development of a viral vector derived from *Prunus necrotic ringspot virus* (PNRSV), a widespread fruit tree virus that is endemic in all *Prunus* fruit production countries and regions in the world. We show that the modified PNRSV vector, harbouring the sense‐orientated target gene sequence of 100‐200 bp in length in genomic RNA3, could efficiently trigger the silencing of a transgene or an endogenous gene in the model plant *Nicotiana benthamiana*. We further demonstrate that the PNRSV‐based vector could be manipulated to silence endogenous genes in peach such as *eukaryotic translation initiation factor 4E isoform* (*eIF(iso)4E*), a host factor of many potyviruses including *Plum pox virus* (PPV). Moreover, the *eIF(iso)4E*‐knocked down peach plants were resistant to PPV. This work opens a potential avenue for the control of virus diseases in perennial trees via viral vector‐mediated silencing of host factors, and the PNRSV vector may serve as a powerful molecular tool for functional genomic studies of *Prunus* fruit trees.

## Introduction

Perennial woody fruit trees are characteristic of a long life cycle, large sizes and recalcitrant to genetic transformation, which severely hinder their genetic and genomic studies and largely affect breeding efforts for fruit improvement and sustainable production. Moreover, the long life span along with vegetative propagation and reproduction, and frequent exchange and transport from various growing regions, makes them easily infected by one or a variety of viral pathogens. These virus diseases are extremely difficult to control and cause severe economic losses. Due to lack of genetic resistance, cross‐protection has been extensively used to control some of these viral diseases in many countries (Ziebell and Carr, 2010). In cross‐protection, a mild isolate of the virus resulting either from natural mutations or engineered modifications is pre‐inoculated for protection of crops against subsequent infection by a severe strain of the same virus. The most successful example of cross‐protection is the control of *Citrus tristeza virus* (CTV), a member of the *Closteroviridae* that is responsible for devastating losses to citrus production all over the world (Bar‐Joseph *et al*., 1989; Dawson and Folimonova, 2013; Dawson *et al*., 2015). Another notorious viral pathogen is *Plum pox virus* (PPV), which causes the disease called sharka in Europe (García *et al*., 2014; Ilardi and Di Nicola‐Negri, 2011; Ilardi and Tavazza, 2015; Rimbaud *et al*., 2015). This virus belongs to the family Potyviridae and causes the most devastating disease in stone fruits. In this case, no suitable natural genetic resistance or mild strains have been found to effectively control it (García *et al*., 2014; Ilardi and Di Nicola‐Negri, 2011; Kegler *et al*., 1998; Martínez‐Gómez *et al*., 2000). Pathogen‐derived resistance (PDR) to PPV has been developed in plum, a natural host of PPV, by genetic engineering (García *et al*., 2014; Ilardi and Di Nicola‐Negri, 2011; Ravelonandro *et al*., 2014; Sochor *et al*., 2012). This type of resistance operates through RNA silencing, which mediates specific degradation of viral RNA (Hily *et al*., 2005; Ravelonandro *et al*., 1997; Scorza *et al*., 2001). Another potential approach for the control of viral diseases is to mutate or silence host factors that are required for viral infection to develop inheritable recessive resistance (Sanfaçon, 2015; Wang, 2015; Wang and Krishnaswamy, 2012). Previously, eukaryotic translation initiation factor 4E isoform (*eIF(iso)4E*) was shown to be a host factor to PPV in the model plant *Arabidopsis* (Decroocq *et al*., 2006). Recently, we have demonstrated that silencing *eIF(iso)4E* in transgenic plum confers PPV resistance (Wang *et al*., 2013). However, genetic resistance through the transgenic approach is not applicable to many *Prunus* species including peach for which genetic transformation is inefficient or not applicable.

Virus‐induced gene silencing (VIGS) is an RNA silencing‐based technology that may be exploited to silence genes of interest in plants (Becker and Lange, 2010; Dawson and Folimonova, 2013; Dolja and Koonin, 2014; Liu *et al*., 2002; Robertson, 2004; Senthil‐Kumar and Mysore, 2011). Briefly, infection by a virus triggers RNA silencing, a plant innate defence pathway that specifically degrades the viral genome. If the virus is engineered to carry a fragment of a plant gene transcript, RNA silencing would be directed to target this particular endogenous gene. The virus‐derived infectious clone is termed as a viral vector. In the past decade, a number of viral vectors have been developed as a powerful reverse genetic tool for the functional characterization of genes in plants (Ding *et al*., 2006; Igarashi *et al*., 2009; Kumagai *et al*., 1995; Kurth *et al*., 2012; Liu *et al*., 2002, 2016; Ruiz *et al*., 1998; Wang *et al*., 2016; Yamagishi *et al*., 2016; Zhang *et al*., 2010). However, the majority of the published VIGS vectors have a host range limited to some herbaceous species, and very few can be used for functional genomic studies of allogamous woody perennials and vines.

Here, we report the development of a viral vector derived from a *Prunus necrotic ringspot virus* (PNRSV) isolate. This widespread fruit tree virus, belonging to the genus *Ilarvirus* in the family *Bromoviridae* and having a viral genome consisting of three RNA segments (Fauquet *et al*., 2005), does not induce severe symptoms in its natural *Prunus* hosts (Cui *et al*., 2012, 2015). We demonstrate that when the PNRSV vector was modified to contain a 100‐200‐bp fragment of a target gene transcript in the sense orientation, it could efficiently trigger gene silencing in both the model plant *Nicotiana benthamiana* and natural host peach. We further show that the PNRSV‐based vector could be employed against PPV via knockdown of *eIF(iso)4E*. Taken together our data suggest that VIGS has a great potential for the control of devastating virus diseases in perennial trees and vines, and the PNRSV vector may be employed for functional genomic studies of *Prunus* fruit trees.

## Results

### PNRSV exhibits properties of a potential VIGS vector in peach

**Figure 1:**
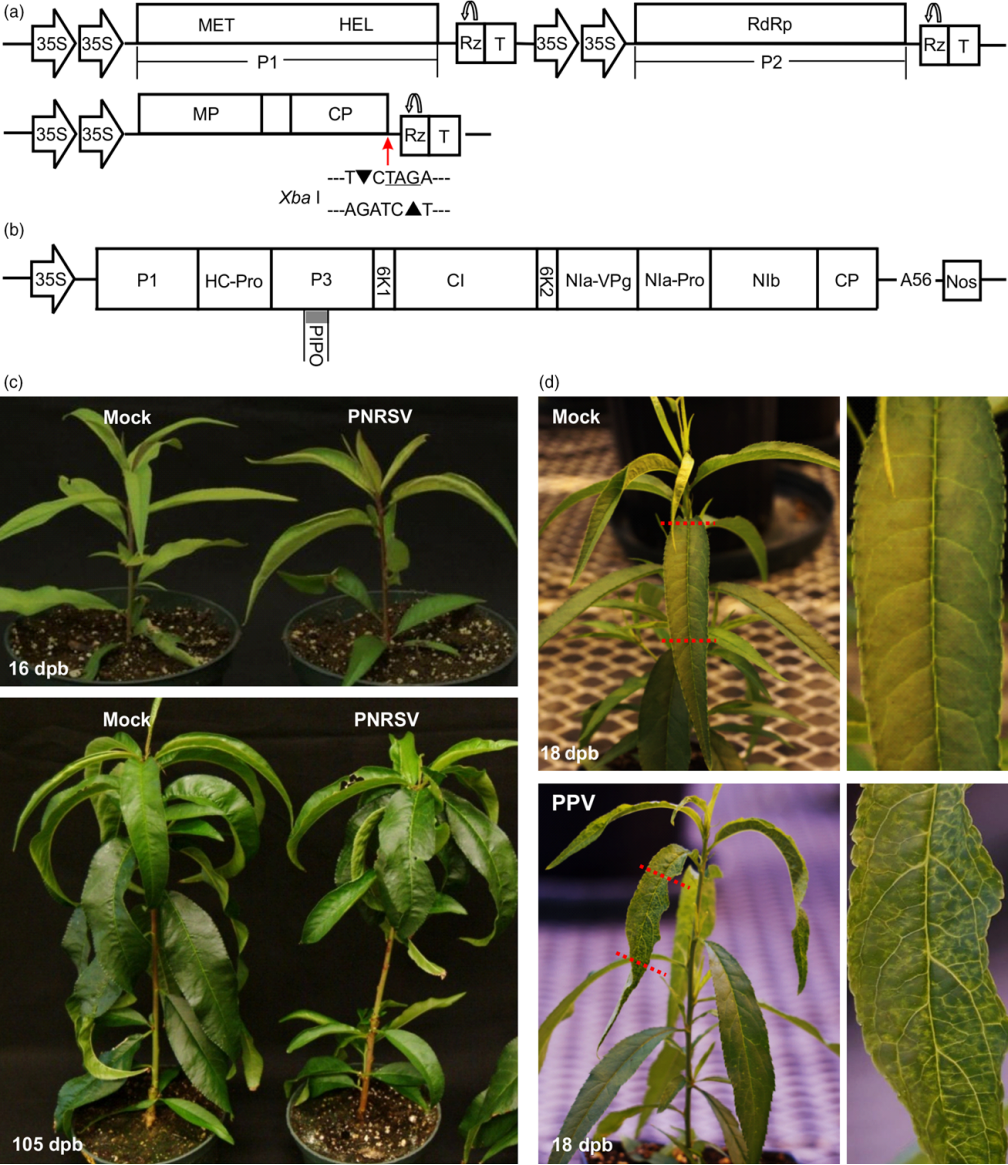
Pathogenicity of the PNRSV‐derived vector and the PPV infectious clone pVPM in peach cv. ‘Loring’. (a) Schematic representation of the PNRSV vector derived from the PNRSV isolate Pch12. Both expression cassettes of genomic RNA1 and RNA2 are integrated into the same pCass4Rz backbone. Monocistronic RNA1 and 2‐encode replicase proteins P1 and P2 respectively. Domains for methyltransferase (MET), helicase (HEL) and RNA‐dependent RNA polymerase (RdRp) are indicated. Dicistronic RNA3 encodes 5′‐proximal movement protein (MP) and 3′‐proximal coat protein (CP), separated by a short intergenic region sequence. The *Xba* I restriction sequence ‘TCTAGA’ (TAG, stop codon of CP gene), located at the junction of CP and 3′ UTR in genomic RNA3, was employed to integrate foreign inserts for construction PNRSV‐based VIGS vectors. The self‐cleavage of ribozyme (Rz) is indicated by a bent arrow; T, 35S terminator. (b) Schematic representation of the infectious cDNA clone of the PPV isolate VPM. The expression cassette of full‐length PPV genomic RNA is integrated into a mini‐binary vector pCB301 backbone. 11 mature proteins encoded by PPV are shown. (c) Disease response induced by the PNRSV vector in ‘Loring’. At 16 days post bombardment (dpb), ‘Loring’ plants infected by PNRSV showed mild distortion and chlorosis symptoms on upper leaves. No visually detectable phenotypic differences were observed among PNRSV‐infected and mock plants at 105 dpb. (d) PPV induced severe vein yellowing, mosaic and distortion symptoms in ‘Loring’ plants at 18 dpb. The images on the right side were magnified from the portion of the corresponding leaf on the left side.

Our recent studies have shown that PNRSV is highly endemic in stone fruit trees in the Niagara Fruit Belt, Canada or different provinces/regions in China, usually without causing obvious phenotypic symptoms in PNRSV‐positive *Prunus* trees (Cui *et al*., 2012, 2015). In addition, PNRSV does not encode any known gene‐silencing suppressors. Moreover, the cDNA clones consisting of three T‐DNA plasmids carrying cDNAs of three full‐length genomic RNA segments of the PNRSV isolate Pch12 were constructed and shown to be infectious in peach cv. ‘Loring’ (Cui *et al*., 2013). Consequently, this PNRSV isolate was considered a suitable candidate to be developed as a VIGS vector for the characterization of gene functions as well as silencing a host factor of PPV against the devastating sharka disease in peach. However, the infection efficiency of the PNRSV infectious clones was about 30% in peach (Cui *et al*., 2013), which is low as a VIGS vector. To improve this deficiency, we modified the system by integrating both RNA1 and RNA2 expression cassettes into the same binary vector. The resulting construct is designated as pCaRNA1&2 (Figure 1a), and expected to allow for the simultaneous expression of both viral genomic RNA1 and RNA2 in the same agro‐transfected cells. To confirm the infectivity of the modified PNRSV clones, cotyledons of cucumber cv. ‘Straight Eight’ were co‐infiltrated with a mixture of agrobacterial cultures harbouring T‐DNA plasmids pCaRNA1&2 and pCaRNA3. Cucumber plants inoculated with original T‐DNA constructs (pCaRNA1, pCaRNA2 and pCaRNA3) were used as a positive control. At 10 days post agroinfiltration (dpai), all cucumber plants, either inoculated with modified or original PNRSV constructs, exhibited similarly severe necrosis and ringspot on newly emerging leaves (Figure S1), demonstrating the co‐placement of genomic RNA1 and RNA2 expression cassettes into the same binary vector had no impact on viral viability.

To determine the infectivity efficiency of the modified PNRSV vectors in peach, eight ‘Loring’ seedlings were biolistically inoculated with two plasmids pCaRNA1&2 and pCaRNA3 mixed in equimolar ratio. At 16 days post bombardment (dpb), six out of eight ‘Loring’ plants showed mild symptoms (very slight chlorosis and distortion) in upper leaves (Figure 1c), consistent with those reported previously (Cui *et al*., 2013). These mild symptoms lasted for 3–5 days and disappeared. This experiment was repeated three times, and similar results were observed. The infectivity of the modified PNRSV vectors is about 75% (14 of 19) in peach, which is significantly higher than ~30% of the previously constructed PNRSV vectors (Cui *et al*., 2013). Therefore, the modified PNRSV vectors were used for the experiments hereafter.

For PPV challenging test in this study, we also constructed a PPV infectious clone derived from a Canadian isolate VPM (PPV‐D strain) (Figure 1b), and the infectivity of pVPM was tested using biolistic bombardment. At 18 dpb, severe symptoms such as altered appearance of venation, yellowing, mosaic and distortion were observed in newly emerging leaves in all 10 pVPM‐inoculated ‘Loring’ seedlings (Figure 1d). Clearly, PPV was indeed much more aggressive than PNRSV in peach (Figure 1d,e), stimulating us to consider if the PNRSV‐based vector can be manipulated to control devastating PPV in stone fruits.

### *N. benthamiana*, is a latent herbaceous host of PNRSV, and may be used to assess the suitability of PNRSV as a VIGS vector

**Figure 2:**
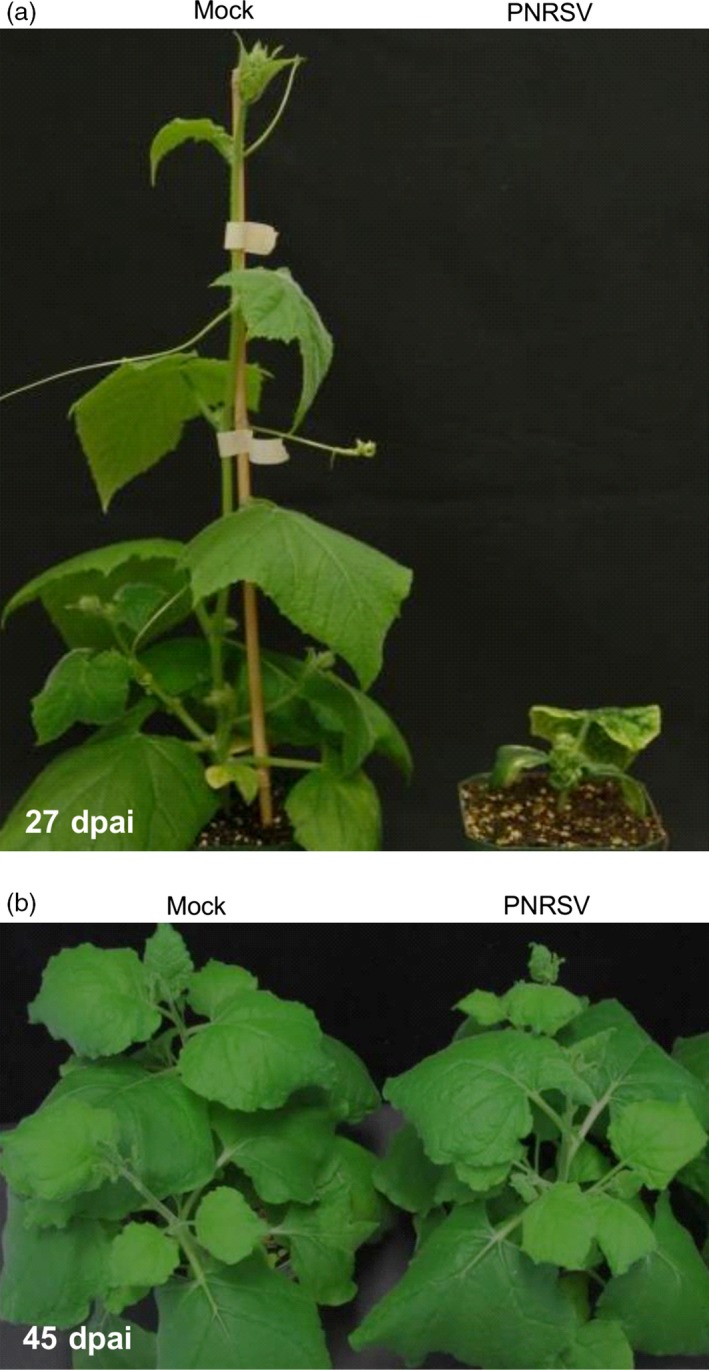
Disease responses triggered by the PNRSV vector in herbaceous hosts *Cucumis sativus* and *Nicotiana benthamiana*. (a) PNRSV‐infected cucumber plants showed extremely dwarfed and necrotic symptoms at 27 days post agroinfiltration (dpai). (b) No visible symptoms were observed in PNRSV‐infected *N. benthamiana* plants at 45 dpai.

As preparation of peach seedlings takes considerable time (approximately 3 months), an alternative herbaceous host of PNRSV was needed for the initial evaluation and optimization of the PNRSV‐based VIGS vectors. We first considered *Cucumis sativus*, a widely used herbaceous diagnostic host. Unfortunately, PNRSV‐infected cucumber plants displayed severe virus‐infected phenotypes, such as ringspot, necrosis and small stature (Figure 2a). Such severe symptoms would mask the gene‐silencing phenotype targeted by PNRSV‐based VIGS vector. So, selection of an appropriate virus–host system where viral symptoms are latent or mild was necessary (Senthil‐Kumar and Mysore, 2011). Another commonly used herbaceous host *N. benthamiana* was tested. DAS‐ELISA assay showed that PNRSV did infect this model plant, but infection did not induce any visually detectable symptoms in all 20 *N. benthamiana* plants inoculated with PNRSV over an extended observation time (i.e. 45 dpai) (Figure 2b). Consequently, *N. benthamiana*, as a latent herbaceous host of PNRSV, was chosen as an ideal host to assess if PNRSV can be used as a VIGS vector.

### PNRSV‐based vectors, harbouring 100‐200‐bp foreign inserts in the sense orientation could trigger silencing of the target genes in *N. benthamiana*

The *PDS* gene encoding phytoene desaturase, a key enzyme in carotenoid biosynthesis, has been widely used as a reporter gene for VIGS test in a variety of plants, as silencing *PDS* can result in a characteristic photo‐bleaching phenotype due to the loss of chlorophyll (Agüero *et al*., 2012; Kurth *et al*., 2012; Ratcliff *et al*., 2001; Ruiz *et al*., 1998; Senthil‐Kumar *et al*., 2007). Therefore, we also tested if PNRSV could silence the *PDS* gene as a VIGS vector in *N. benthamiana*. Given that different viruses could tolerate foreign inserts in a particular range of sizes, a series of PNRSV‐derived vectors, harbouring the *PDS* fragments of varied sizes in the antisense or sense orientation, were constructed. For construction of these PNRSV‐based vectors, the unique restriction *Xba* I sequence ‘TCTAGA’ (containing TAG the stop codon of the *CP* gene) in genomic RNA3 was used to integrate foreign inserts (Figure 1a). This would warrant that the foreign insert in genomic RNA3 did not affect the coding region of *CP*.

**Figure 3:**
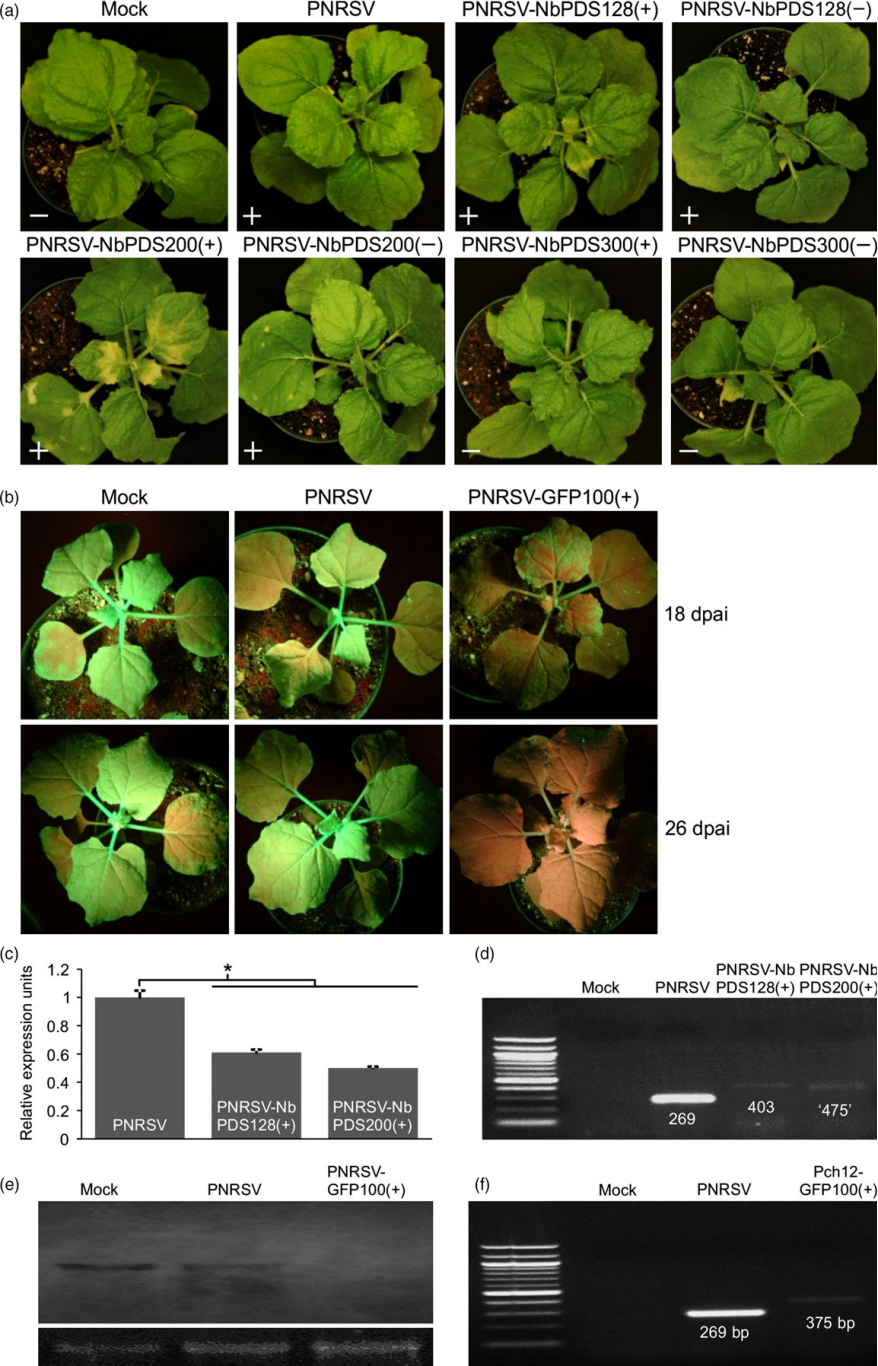
Silencing of *PDS* or *GFP* mediated by the PNRSV‐based vectors, in wild‐type or *gfp*‐transgenic *Nicotiana benthamiana* plants. (a) *PDS*‐silenced *N. benthamiana* plants inoculated with different PNRSV‐based constructs harbouring foreign inserts of varied sizes in the sense or antisense orientation. At 20 days post agroinfiltration (dpai), *N. benthamiana* plants either agroinfiltrated with PNRSV‐NbPDS100(+) or PNRSV‐NbPDS200(+) showed the typical photo‐bleaching phenotype in the upper leaves, resulting from the reduction in the expression level of *PDS*. Symbols ‘+’ and ‘−’ represent the presence and absence of PNRSV by DAS‐ELISA analysis respectively. (b) The PNRSV‐based construct triggers systemic silencing of *GFP* in *gfp*‐transgenic *N. benthamiana* (16c). The green/red fluorescence in ‘16c’ plants inoculated with wtPNRSV or PNRSV‐GFP100(+) was excited under UV illumination. At 18 dpai, red fluorescence (chlorophyll autofluorescence), representing loss of *GFP*, was observed in upper leaves of the ‘16c’ plants inoculated with PNRSV‐GFP100(+). Subsequently, the *GFP* gene was silenced in the entire plants at 26 dpai. No GFP‐silenced phenotype was observed in ‘16c’ plants either inoculated with wtPNRSV or buffer‐treated (Mock) at all time points. (c) The relative expression level of *PDS* in *N. benthamiana* plants agroinfiltrated with PNRSV‐NbPDS100(+), PNRSV‐NbPDS200(+) or wtPNRSV control was determined by qRT‐PCR. Actin transcript levels were determined as an internal control. The leaf tissues showing photo‐bleaching were sampled for qRT‐PCR analysis at 20 dpai. Error bars denote standard errors of three independent biological replicates. Statistically significant differences, determined by an unpaired two‐tailed Student's *t* test, are indicated by brackets. *0.01 < *P *<* *0.05. (d) Conventional RT‐PCR analysis of genetic stability of the foreign insert in the recombinant virus using primers RNA3‐CP‐F/RNA3‐3′UTR‐R. The predicted sizes of the RT‐PCR products derived from *N. benthamiana* plants infected with wtPNRSV, PNRSV‐NbPDS100(+) and PNRSV‐NbPDS200(+) are 269‐, 403‐ and 475‐bp respectively. However, the fragment amplified from plants inoculated with PNRSV‐NbPDS200(+) was approximately 400 bp but not 475 bp in length, indicating that the 200‐bp PDS fragment in recombinant virus was partially lost via a yet unidentified mechanism. (e) Northern blot analysis of the mRNA expression level of *GFP* in ‘16c’ plants infected with wtPNRSV, PNRSV‐GFP100(+) or buffer‐treated (mock) at 26 dpai. Ribosomal RNA (28S), stained with ethidium bromide, was used as a loading control. (f) Conventional RT‐PCR analysis of genetic stability of the foreign insert in the recombinant virus using primers RNA3‐CP‐F/RNA3‐3′UTR‐R. Consistent with the predicted sizes, RT‐PCR products obtained from ‘16c’ plants infected with wtPNRSV and PNRSV‐GFP100(+), were 269 and 375 bp in length respectively.

The resulting pCaRNA3‐derived binary vectors, together with pCaRNA1&2, were delivered into five *N. benthamiana* plants via agroinfiltration. At 20 dpai, the photo‐bleaching phenotype, representing the loss of *PDS*, was observed in the upper leaves of all plants inoculated with either PNRSV‐NbPDS128(+) or PNRSV‐NbPDS200(+) (Figure 3a). Consistently, real‐time qPCR showed that the mRNA expression level of *PDS* in these plants was significantly lower than that in the plants infected with wild‐type PNRSV (Figure 3c). For the convenience of description, ‘wild‐type PNRSV’ is designated as wtPNRSV hereafter. The genetic stability of foreign inserts in these recombinant viruses was evaluated by conventional RT‐PCR using the primer set RNA3‐CP‐F and RNA3‐3′UTR‐R (Table S1). The predicted sizes of RT‐PCR amplification products derived from plants infected with wtPNRSV, PNRSV‐NbPDS128(+) and PNRSV‐NbPDS200(+) were 269‐, 403‐ and 475‐bp respectively. Indeed, the RT‐PCR products of predicted sizes were evident from samples of wtPNRSV‐ and PNRSV‐NbPDS128(+)‐ infected plants. However, the fragment amplified from plants inoculated with PNRSV‐NbPDS200(+) was approximately 400 bp rather than 475 bp in length (Figure 3d), indicating that the 200‐bp *PDS* fragment in PNRSV‐NbPDS200(+) was partially lost. Apparently, PNRSV‐based viral vectors are not suitable for carrying foreign fragments with large sizes. Surprisingly, no *PDS*‐silenced phenotype was observed in plants either inoculated with PNRSV‐NbPDS100(−) or PNRSV‐NbPDS200(−), although the presence of PNRSV in these plants was confirmed by DAS‐ELISA assay (Figure 3a). Clearly, the PNRSV vector carrying the foreign inserts in the sense but not the antisense orientation could efficiently trigger gene silencing in *N. benthamiana*. Neither the *PDS*‐silenced phenotype nor the presence of PNRSV were identified in plants either inoculated with PNRSV‐NbPDS300(+) or PNRSV‐NbPDS300(−) (Figure 3a), further indicating that the PNRSV vector could not tolerate large foreign inserts. Collectively, these data suggested that the PNRSV‐based vector, harbouring a foreign insert of 100‐200 bp in the sense orientation could efficiently silence the corresponding gene in *N. benthamiana*.

To further test if the PNRSV vector has the ability to silence a transgene in plants, we used the *gfp*‐transgenic *N. benthamiana* line (16c) (Ruiz *et al*., 1998). For this assay, we first constructed the vector pCaRNA3‐GFP100(+) containing a 100‐bp *GFP* fragment in the sense orientation in genomic RNA3. The PNRSV‐GFP100(+) plasmid was then delivered into eight ‘16c’ plants via agroinfiltration. At 18 dpai, obvious red fluorescence (chlorophyll autofluorescence) rather than green fluorescence was observed to expand along venation in upper leaves of all eight ‘16c’ plants under UV light illumination, reflecting silencing of the *GFP* gene (Figure 3b). The green fluorescence was barely detectable in the entire plant at 26 dpai, suggesting that the *GFP* gene was silenced to a great extent (Figure 3b). This was further confirmed by the result of northern blot showing the undetectable mRNA level of *GFP* in the silenced plant mediated by the VIGS vector (Figure 3e). In these plants, the 100‐bp *GFP* fragment in genomic RNA3 was genetically stable, which was verified by conventional RT‐PCR using the primer set RNA3‐CP‐F and RNA3‐3′UTR‐R (Figure 3f). As a control, no *GFP*‐silenced phenotype was observed in the ‘16c’ plants either inoculated with wtPNRSV or buffer treated (mock) at 18 or 26 dpai. Consistently, northern blot analysis showed no obvious differences in GFP mRNA expression among these plants at 26 dpai (Figure 3e).

### The PNRSV‐based vector has the ability to silence an endogenous gene in peach

**Figure 4:**
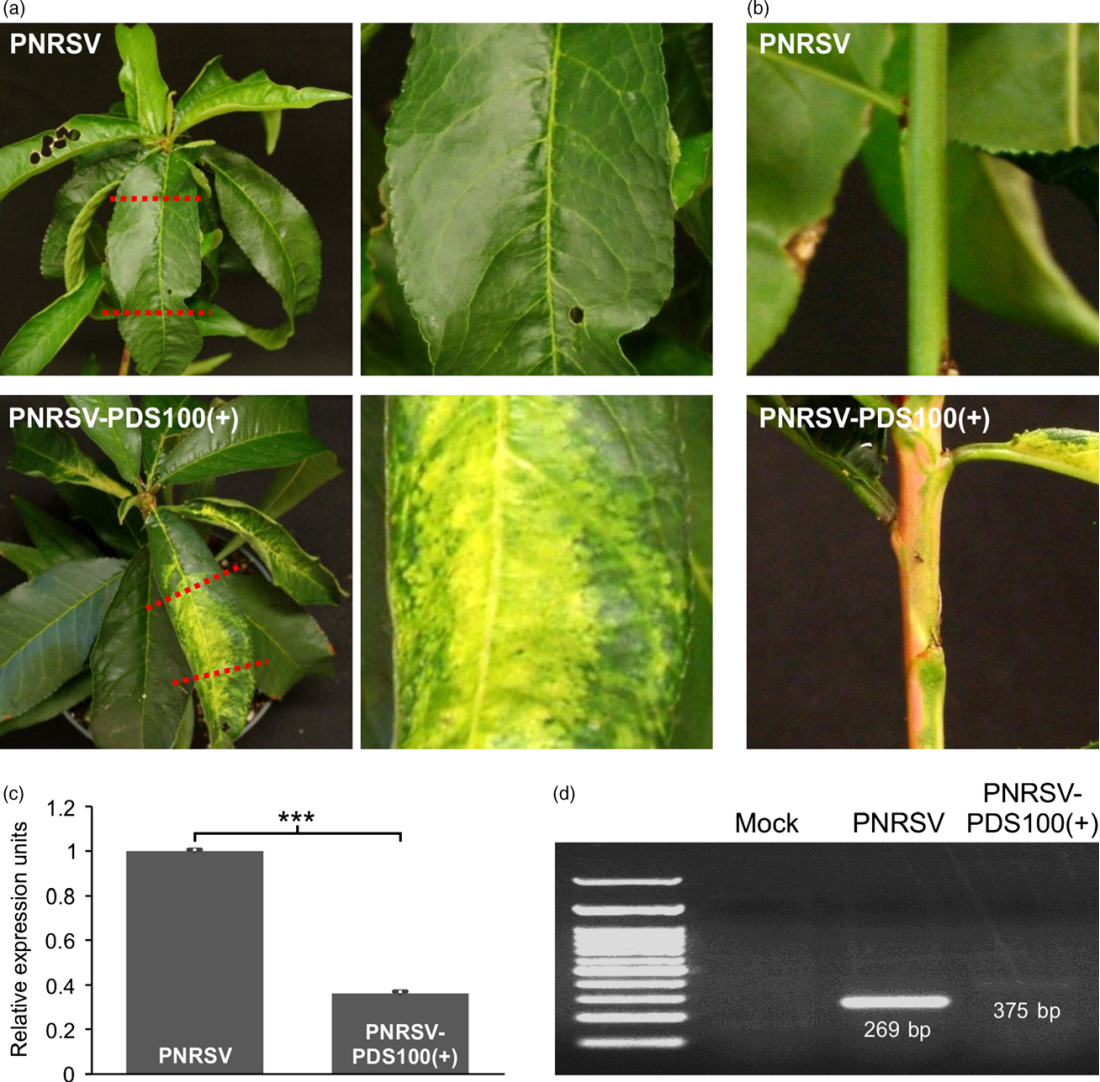
Down‐regulation of *PDS* in peach by a modified PNRSV‐based vector. (a, b) At 60 days post bombardment (dpb), ‘Loring’ plants infected with PNRSV‐pchPDS100(+) displayed typical photo‐bleaching phenotype in upper leaves (a) and stems (b), indicating the down‐regulation of the *PDS* gene in peach. The images on the right side (a) were magnified from the portion of the corresponding leaf on the left side. (c) The relative expression level of *PDS* in ‘Loring’ plants infected with wtPNRSV or PNRSV‐pchPDS100(+). qRT‐PCR was carried out with the *Actin* transcript levels as an internal control. The leaf tissues showing photo‐bleaching were sampled for qRT‐PCR analysis at 60 dpb. Error bars denote standard errors of three independent biological replicates. Statistically significant differences, determined by an unpaired two‐tailed Student's *t* test, are indicated by brackets. ***P <* *0.001. (d) Conventional RT‐PCR analysis of genetic stability of the foreign insert in recombinant viruses using the primer set RNA3‐CP‐F/RNA3‐3′UTR‐R. PCR products of the predicted sizes 269 and 375 bp were amplified from ‘Loring’ plants infected with wtPNRSV and PNRSV‐pchPDS100(+) respectively.

VIGS has been proven to be a powerful tool for the functional characterization of genes in model and crop plants (Baulcombe, 1999; Becker and Lange, 2010; Burch‐Smith *et al*., 2004; Senthil‐Kumar and Mysore, 2011; Waterhouse and Helliwell, 2003). In recent years, several fruit tree‐infecting viruses, that is, *Citrus leaf blotch virus* (CLBV), *Grapevine leafroll‐associated virus*‐2 (GLRaV‐2) and *Apple latent spherical virus* (ALSV) have been developed into VIGS vectors to characterize gene function in a few species of fruit trees (Agüero *et al*., 2012, 2014; Kawai *et al*., 2016; Kurth *et al*., 2012; Sasaki *et al*., 2011; Yamagishi *et al*., 2016). However, no suitable VIGS vectors have been established for peach. To test whether the PNRSV‐based vectors could be used in peach, the vector pCaRNA3‐pchPDS100(+), harbouring a peach 100‐bp *PDS* fragment in the sense orientation in genomic RNA3, was constructed. The PNRSV‐pchPDS100(+) constructs were biolistically introduced into eight peach ‘Loring’ seedlings. At 60 dpb, five out of eight plants are tested to be PNRSV positive by DAS‐ELISA assay. Also, the typical photo‐bleaching phenotype, resulting from down‐regulation of the *PDS* gene, was observed in stems and upper leaves in all the five plants infected by the modified VIGS vector (Figure 4a,b). Consistently, the mRNA expression level in these plants was significantly lower than that in wtPNRSV‐infected plants (Figure 4c). The similar *PDS*‐silenced phenotype was also observed in the subsequent growing cycle after 2‐month cold treatment.

The genetic stability of the foreign insert in the recombinant virus was investigated using conventional RT‐PCR with the primer set RNA3‐CP‐F/RNA3‐3′UTR‐R flanking the foreign insert in genomic RNA3. As expected, RT‐PCR yielded 375‐bp and 269‐bp products from samples infected with PNRSV‐PchPDS100(+) and wtPNRSV respectively (Figure 4d). These data suggest that the PNRSV‐based vector can be used to knock down endogenous genes in peach, a perennial woody plant.

### Silencing of *eIF(iso)4E* leads to resistance to PPV in peach

In addition to their uses in studying gene function, viral vectors may have potential against pathogens or invertebrate pests via RNAi‐enabled vaccination (Dawson and Folimonova, 2013; Kurth *et al*., 2012; Nicaise, 2014). Here, we attempted to utilize the PNRSV‐based vector to control sharka disease by knocking down the *eIF(iso)4E* gene in peach. Previously, it was shown that *eIF(iso)4E* was required for PPV infection either in *Arabidopsis* or plum (*Prunus domestica* L.) (Decroocq *et al*., 2006; Wang *et al*., 2013). Sequence analysis showed that *eIF4E* and its isoform *eIF(iso)4E* of peach shared about 56% sequence identity at the nt level (Figure S3). To avoid non‐specific silencing of the *eIF4E* gene, the N‐terminus of *eIF(iso)4E*, relatively divergent with that of *eIF4E* (Figure S3), was selected to construct the gene‐silencing vector pCaRNA3‐eIFiso4E120(+).

**Figure 5:**
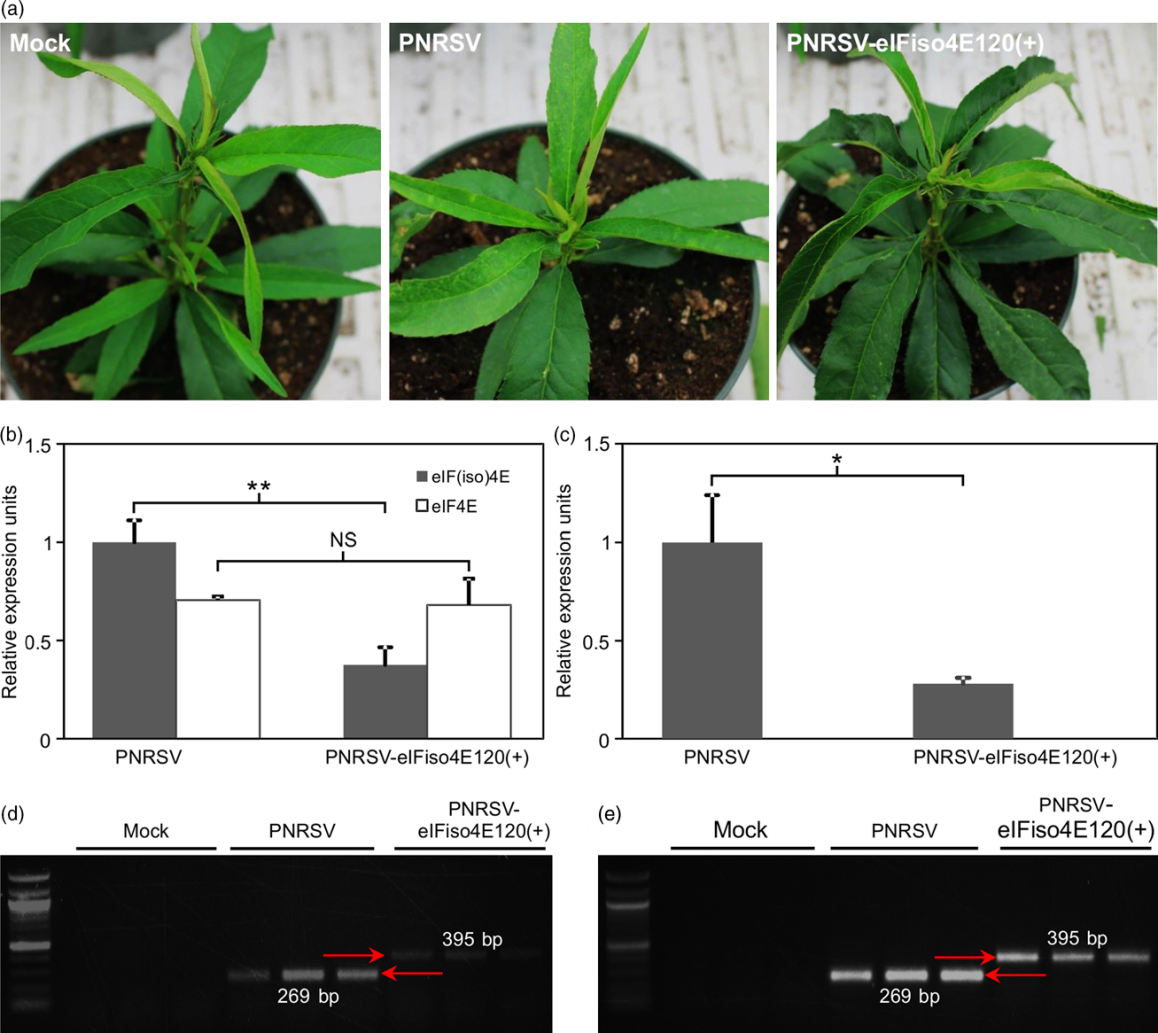
The PNRSV‐based vector has the ability to knock down the expression of the *eIF(iso)4E* gene in peach. (a) The phenotype of PNRSV‐mediated knockdown of *eIF(iso)4E* in peach. At 22 days post bombardment (dpb), mild chlorosis and distortion symptoms were observed in plants either infected with wtPNRSV or PNRSV‐eIFiso4E120(+). Except these typical symptoms induced by PNRSV, no other physiological abnormality was observed among these plants. (b, c) The relative expression level of *eIF(iso)4E* or *eIF4E* in ‘Loring’ plants infected with wtPNRSV or PNRSV‐eIFiso4E120(+) at 16 (b) and 22 dpb (c). qRT‐PCR was performed, and the *Actin* transcript levels were used as an internal control. Error bars denote standard errors of three independent biological replicates. Statistically significant differences, determined by an unpaired two‐tailed Student's *t* test, are indicated by brackets. NS, no significant differences; *P <* *0.05; **P <* *0.01. (d, e) Conventional RT‐PCR analysis of genetic stability of the foreign insert in recombinant viruses using the primer set RNA3‐CP‐F/RNA3‐3′UTR‐R at 16 (d) and 90 dpb (e). PCR products of the predicted sizes 269 and 395 bp were amplified from ‘Loring’ plants infected with wtPNRSV and PNRSV‐eIFiso4E120(+), respectively.

To determine whether pCaRNA3‐eIFiso4E120(+) could efficiently and specially down‐regulate the expression of the *eIF(iso)4E* gene in peach, the recombinant and wtPNRSV clones were biolistically introduced into peach seedlings. At 16 dpb, mild chlorosis and distortion symptoms in upper leaves, indicative of PNRSV infection, were observed in four and three out of five peach plants inoculated with wtPNRSV and recombinant PNRSV‐eIFiso4E120(+) respectively (Figure 5a). These mild symptoms lasted for 3–5 days and then diminished completely, consistent with our previous observations (Cui *et al*., 2013). Except the transient typical symptoms induced by PNRSV, no other physiological abnormity was observed among these plants (Figure 5a). The mRNA expression level of *eIF(iso)4E* or *eIF4E* was detected by qRT‐PCR at 16 or 22 dpb. The results revealed that the relative amount of *eIF(iso)4E* transcripts in ‘Loring’ plants infected with PNRSV‐eIFiso4E120(+) was significantly lower than that of wtPNRSV‐infected plants, and no significant difference of the *eIF4E* expression level was observed between PNRSV‐eIFiso4E120(+)‐ and wtPNRSV‐infected plants (Figure 5b). At 22 dpb, the difference in the expression of *eIF(iso)4E* in these plants was further magnified (Figure 5c). The foreign fragment in the recombinant virus was shown to be genetically stable in the infected plants at 16 or 90 dpb (Figure 5d,e). Altogether, these data demonstrated that the construct PNRSV‐eIFiso4E120(+) could efficiently and specially down‐regulate the expression of *eIF(iso)4E* in peach.

**Figure 6:**
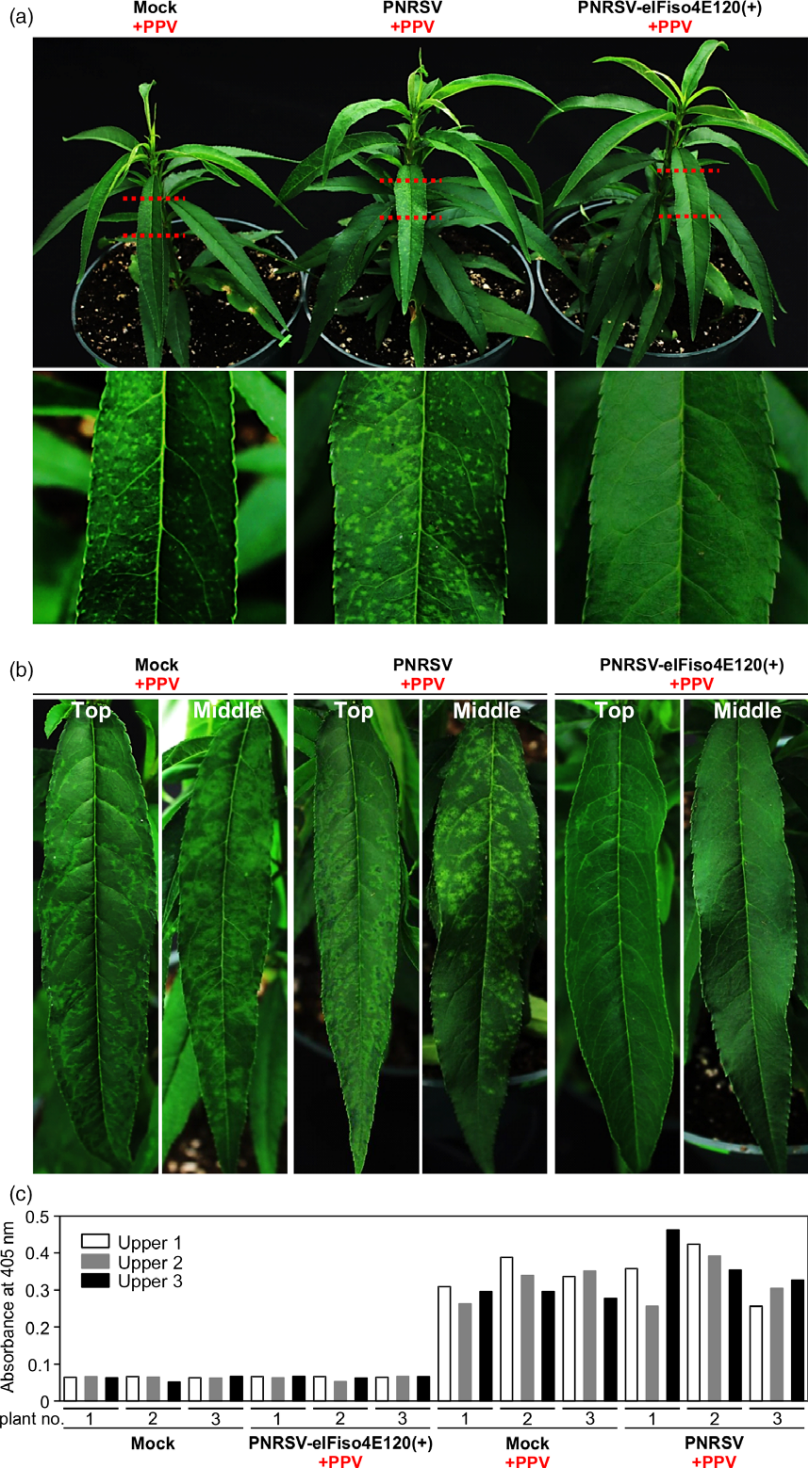
Knockdown of *eIF(iso)4E* expression confers PPV resistance in peach. (a, b) Disease response induced by PPV in *eIF(iso)4E*‐silenced and wtPNRSV‐infected ‘Loring’ plants. At 22 days post bombardment with wtPNRSV or PNRSV‐eIFiso4E120(+), the infected ‘Loring’ plants were biolistically challenged with PPV. Also, mock plants were bombarded with PPV and used as a positive control. 16 days later, severe symptoms such as chlorotic spots and venation chlorosis were observed in all mock/PPV and wtPNRSV/PPV plants. In contrast, PNRSV‐eIFiso4E120(+)/PPV plants did not show any PPV symptom (a). Until 25 days later, no obvious symptoms were observed in PNRSV‐eIFiso4E120(+)/PPV plants (b). (c) Detection of PPV by semi‐quantitative DAS‐ELISA at 16 days post‐inoculation with PPV.

For the PPV resistance assay, five and four peach seedlings infected with wtPNRSV and PNRSV‐eIFiso4E120(+), respectively, were obtained essentially as described above. At 22 dpb, plants were biolistically inoculated with the PPV infectious clone pVPM, and the five mock‐treated plants bombarded with PPV were used as a control. Severe symptoms such as chlorotic spots and chlorosis along with venation, indicative of an early infection of PPV, were observed in all mock/PPV and wtPNRSV/PPV plants at 16 days post PPV‐inoculation (Figure 6a). In contrast, all four PNRSV‐eIFiso4E120(+)/PPV plants did not show any PPV symptoms (Figure 6a). This phenotype was further confirmed by detecting the PPV accumulation level in these plants using a semi‐quantitative DAS‐ELISA assay (Figure 6c). Until 25 days later, no obvious symptoms were detected in PNRSV‐eIFiso4E120(+)/PPV plants (Figure 6b). Taken together, these data demonstrated that the PNRSV‐based construct PNRSV‐eIFiso4E120(+) could efficiently down‐regulate *eIF(iso)4E* expression, leading to resistance to PPV.

## Discussion

In this study, we developed a PNRSV‐based viral vector suitable for the characterization of gene function in peach. Most importantly, we show that the PNRSV‐based viral vector could be manipulated to silence *eIF(iso)4E*, a host factor of PPV, to control sharka disease in peach and likely in other *Prunus* spp. To our best knowledge, this was the first report of the successful construction and application of a viral vector in a perennial *Prunus* plant. It is worth mentioning that recently, a *Tobacco rattle virus* (TRV)‐based vector has been successfully employed to evaluate gene function related to fruit traits in peach through agroinfiltration into the flesh of ripening peach fruits (Bai *et al*., 2016; Zhou *et al*., 2015). However, *Prunus* spp. are not the compatible hosts of TRV, largely limiting its application as a genomics study tool. The availability of the PNRSV‐based vector makes it possible to silence genes of interest and control viral diseases via silencing a particular host factor gene in peach trees. The importance of this vector is significant as it offers an alternative approach to genetic transformation which is still problematic for peach. Although PNRSV has been historically deemed as a pathogenic virus of *Prunus* spp. (Pallas *et al*., 2012), the isolate Pch12 that has been developed as a viral vector in this study caused mild symptoms on peach trees which lasted for only a few days and then disappeared (Figure 1c), and in the field conditions, Pch12‐infected trees did not show any disease phenotype either (Cui *et al*., 2012). In addition, the virus is highly endemic in all the *Prunus* fruit production countries and regions in the world (Cui *et al*., 2015; Pallas *et al*., 2012). Consequently, these characteristics open up the possibility for the practical application of the PNRSV‐based vector.

Application of PNRSV as a VIGS vector requires the insertion of foreign sequences into the PNRSV genome at positions that do not affect viral infectivity. However, the compact structure of the tripartite RNA genome of PNRSV only encodes four genes, and each of them is indispensable for viral replication, movement or packaging. To simplify genetic engineering, we used an *Xba* I restriction sequence containing stop codon and located right after the CP sequence and before the 3′ UTR in genomic RNA3, for insertion of target gene fragments (Figure 1a). This strategy would not affect the CP coding sequence and the 3′ UTR of genomic RNA3. It is generally believed that the 3′ UTR of alfamo‐ and ilaviruses provides specific binding sites to the CP for the invading virus to initiate infection (Aparicio *et al*., 2003; Bol, 2005; Jaspars, 1999). In addition, along with viral multiplication *in planta*, the modified PNRSV would produce genomic RNA3 and subgenomic RNA4, both of which contain the foreign insert. This would allow for the accumulation of greater amounts of foreign fragment‐derived siRNAs and enhance the gene‐silencing efficiency. Foreign fragment‐derived siRNAs could be produced through either viral replicative intermediate dsRNA which is cleaved by Dicer‐like (DCL) ribonucleases to produce siRNA (Ding and Voinnet, 2007; Llave, 2010) or dsRNA catalysed by plant RNA‐dependent RNA polymerases using viral single‐stranded RNA as a template (Donaire *et al*., 2008).

For spherical plant viruses whose virions are in a similar size range (25–35 nm), this could limit the packaging capacity of the viral genome (Rao, 2006). These viruses include *Alfalfa mosaic virus* (AMV) and BMV (Kasteel *et al*., 1997; Schmitz and Rao, 1996), the type member of the genera *Alfamovirus* and *Bromovirus* in the family *Bromoviridae* respectively. As mentioned earlier, PNRSV is a member of the *Bromoviridae* family too, and closely related to AMV and BMV. The physical size constraint of virions might explain why the PNRSV‐based vector could not tolerate foreign inserts of large sizes. The similar results were also observed in other spherical viruses such as *Turnip yellow mosaic virus* (TYMV) and *Cucumber mosaic virus* (CMV) (Pflieger *et al*., 2008; Wang *et al*., 2016). Such a limitation precludes the further development of this type of viruses as an expression vector. The use of small inserts for PNRSV vectors seems efficient in silencing endogenous genes such as *PDS* and *eIF(iso)4E* in peach (Figures 4 and 5) likely due to viral genomic stability, as previously suggested (Lacomme and Hrubikova, 2003; Pflieger *et al*., 2008; Simon and Bujarski, 1994). In addition, the use of small inserts could decrease the likelihood of unintentional cross‐silencing of related genes (Pflieger *et al*., 2008). However, the PNRSV‐based system did not highly efficiently silence the *PDS* gene in *N. benthamiana* but did silence the *GFP* transgene in this model herb plant (Figure 3a). This could attribute to the less‐ideal *PDS* fragment selected as a silencing target. Further attempts should be made to improve the efficiency of gene silencing of the PNRSV‐based system. For example, it should be investigated whether integration of an inverted‐repeat fragment of target genes into the PNRSV vector could enhance gene silencing, as previously demonstrated for TYMV and TMV vectors (Lacomme and Hrubikova, 2003; Pflieger *et al*., 2008).

Sharka disease, firstly described on plums in Bulgaria in 1917 (Atanasoff, 1933), has been believed to be the most devastating disease in *Prunus* fruits (Cambra *et al*., 2006; Wang *et al*., 2006). Genetic engineering has been pursued by numerous laboratories worldwide to generate PPV resistance in some *Prunus* fruits such as plum (García *et al*., 2014; Ilardi and Di Nicola‐Negri, 2011; Ravelonandro *et al*., 2014). However, it is almost impossible to adapt these strategies to achieve PPV resistance in some other *Pruus* spp. such as peach, because genetic transformation is technically challenging for them. Also, public concerns still strongly prevent commercialization of genetically modified foods, particularly fruits. Virus‐based vectors may bypass this obstacle as these vectors can deliver some nucleic acid/protein vaccines into plants to counteract diseases or pests for perennial crops (Dawson and Folimonova, 2013; Kurth *et al*., 2012). It is worth mentioning that viral vectors could be an attenuated version of a pathogenic viral pathogen used in cross‐protection, an established technology for disease control for a number of crops including vegetables and fruits for long time (Ziebell and Carr, 2010).

In conclusion, the PNRSV‐based vector reported in this study provides a platform for cost‐effective studies of gene function in peach and closely related species. In addition, the vector could be modified to serve as a nucleic acid ‘vaccine’ for the control of the devastating viral pathogens in stone fruit trees.

## Experimental procedures

### Plant materials and viral isolates

The PNRSV natural host peach (*Prunus persica* cv. ‘Loring’) and experimental hosts cucumber (*C. sativus* cv. ‘Straight Eight’), wild‐type *N. benthamiana* and the constitutively GFP‐expressing *N. benthamiana* line 16c (Ruiz *et al*., 1998) were used in this study for gene silencing or pathogenicity test. For pathogenicity test, all plants were maintained in a greenhouse set to 16 h of light at 23 °C and 8 h of darkness at 20 °C. All plants used for gene‐silencing test were maintained in a growth chamber with a 16/8‐h light/dark cycle at 20 °C. ‘Loring’ peach seedlings were generated as previously described (Cui *et al*., 2013).

Fresh plum leaf tissues infected by PPV (PPV‐D strain) were collected from an experimental orchard at Jordan Station, Ontario, Canada, and used for cloning of the full‐length genome and construction of the infectious cDNA clone. Here, the Canadian PPV isolate was designated as VPM.

### Plasmid construction

As previously described, the infectious cDNA clones of PNRSV isolate Pch12 contained three binary plant expression constructs, which could express the corresponding genomic RNA1, RNA2 and RNA3 of PNRSV once delivered into plant cells via agroinfiltration or biolistic bombardment (Cui *et al*., 2013). For the convenience of description, these three binary constructs were named as pCaRNA1, pCaRNA2 and pCaRNA3 respectively. To optimize infectivity efficiency of the infectious clones, both RNA1 and RNA2 expression cassettes were integrated into the same binary vector. For this, the complete RNA1 expression cassette, including 2 × 35S promoter, the entire cDNAs of RNA1 and termination sequences, was amplified from pCaRNA1 by primers HinIII‐35S‐F/HindIII‐35ST‐R (Table S1). The resulting fragment was treated by *Hind* III, and inserted into *Hind* III/CIP‐treated pCaRNA2 to obtain the construct pCaRNA1&2 (Figure 1a).

Similar with our previous strategy to construct pVPH (Cui and Wang, 2016), the VPM‐derived full‐length cDNA was integrated into the mini‐binary vector pCB301 (Xiang *et al*., 1999) under the control of 35S promoter (Note: the genomic sequence of the PPV isolate was deposited into GenBank database with accession number KU948432). The resulting infectious cDNA clone of PPV was designated as pVPM (Figure 1b). All the constructs in this study were confirmed by sequencing.

For a gene‐silencing test with *PDS* as a reporter gene in *N. benthamiana*, a series of pCaRNA3‐based vectors harbouring different *PDS* fragments of varied sizes in the antisense or sense orientation were constructed. Three primer sets NbPDS128‐F/NbPDS‐R, NbPDS200‐F/NbPDS‐R and NbPDS300‐F/NbPDS‐R (Table S1) were designed to amplify 128‐, 200‐ and 300‐bp fragments of the *N. benthamiana PDS* gene respectively (Figure S2). The resulting fragments were digested with *Xba* I, and ligated into *Xba* I/CIP‐treated pCaRNA3, and the ligated plasmid was transformed into *Escherichia coli* (DH10B strain) competent cells. For colonies’ screening, primers RNA3‐CP‐F and RNA3‐3′UTR‐R (Table S1), homologous and complementary to nt 1683‐1702 and 1932‐1951 of PNRSV genomic RNA3 (GenBank Accession # JN416776), respectively, were used to identify colonies carrying a single copy of the inert and determine the sense/antisense orientation of the foreign insert in pCaRNA3. The resulting six pCaRNA3‐derived constructs were named pCaRNA3‐NbPDS128(+), pCaRNA3‐NbPDS128(−), pCaRNA3‐NbPDS200(+), pCaRNA3‐NbPDS200(−), pCaRNA3‐NbPDS300(+) and pCaRNA3‐NbPDS300(−). Similarly, the vectors pCaRNA3‐NbGFP100(+) and pCaRNA3‐pchPDS100(+) were generated to silence the *GFP* gene in *gfp*‐transgenic *N. benthamiana* and the *PDS* gene in peach respectively.

For the construction of pCaRNA3‐pcheIFiso4E120(+), the *eIF4E* and its isoform *eIF(iso)4E* sequences of *P. persica* were retrieved from GDR database (https://www.rosaceae.org/) and subjected for sequence alignment using CLUSTALX 1.8 (Figure S3). The N‐terminus of *eIF(iso)4E*, relatively divergent with the corresponding region of *eIF4E*, was selected to construct pCaRNA3‐pcheIFiso4E120(+) (Figure S3), essentially as described above.

### Agroinfiltration and biolistic bombardment

Seedlings of *N. benthamiana* (3~4 leaf stage) and cucumber ‘Straight Eight’ with fully expanded cotyledons were used for *Agrobacterium*‐mediated transient expression. Briefly, each of binary vectors was transformed into *Agrobacterium tumefaciens* GV3101 via electroporation. Agrobacteria were grown overnight in Luria–Bertani medium containing kanamycin at 100 μg/mL, rifampicin at 20 μg/mL and gentamycin at 20 μg/mL. The agrobacterial cells were harvested by centrifugation, and then resuspended in infiltration buffer (10 mm MgCl_2_, 10 mm MES and 150 μm acetosyringone). After incubation for 2 h at room temperature, the culture was adjusted to OD_600_ (1.0). Two agrobacterium cultures, harbouring pCaRNA1&2 and pCaRNA3‐based vectors, respectively, were mixed with equal volume, and infiltrated into cotyledons of cucumber or completely expanded leaves of *N. benthamiana*.

Peach ‘Loring’ seedlings (3–4 weeks old and approximately 10–15 cm in length) were biolistically inoculated using the Helios Gene Gun System (Bio‐Rad, Hercules, CA). Microcarrier cartridges were prepared with 1.0 μm gold particles coated with the equally mixed plasmids at a DNA loading ratio of 2 μg/mg and a microcarrier loading quantity of 0.5 mg/shooting, according to the manufacturer's instructions. Helium pressure of 160–180 psi were used, and two cartridges were shot onto different leaves per plant.

All agroinfiltration or biolistic bombardment experiments were conducted at least three times (as independent biological replicates).

### DAS‐ELISA and RT‐PCR

After agroinfiltration or biolistic bombardment, PNRSV or PPV in inoculated plants was detected by DAS‐ELISA or RT‐PCR at specific time points indicated. DAS‐ELISA to detect PNRSV was conducted with an ELISA kit (Agdia, Elkhart, IN, U.S.A). To quantify the PPV accumulation level in peach plants, Bradford assay was performed to equal the loading total proteins in crude extracts, and DAS‐ELISA was conducted with an ELISA kit (Agdia). For RT‐PCR, total RNAs were extracted from newly emerging leaves using RNeasy Plant Mini Kit (Invitrogen, Burlington, ON, Canada) and treated with DNase I. The first‐strand cDNA was generated by reverse‐transcription reactions with random hexamer primer (New England Biolabs, Whitby, ON, Canada) and SuperScript III Reverse Transcriptase (Invitrogen). PCR with the primer set RNA3‐CP‐F and RNA3‐3′UTR‐R (Table S1) was carried out to detect the genetic stability of foreign inserts of PNRSV‐based vectors *in planta*.

### qRT‐PCR and northern blot analysis

qRT‐PCR was used to analyse the mRNA expression level of the endogenous genes *PDS*,* eIF4E* or *eIF(iso)4E* in either *N. benthamiana* or peach ‘Loring’ plants inoculated with PNRSV‐based vectors at days indicated. The first‐strand cDNAs were obtained as described above. The expression level of *PDS*,* eIF(iso)4E* and *eIF4E* of peach ‘Loring’ was determined using primer sets pchPDS145F/pchPDS145R, qiso4E‐F/qiso4E‐R and q4E‐F/q4E‐R, respectively (Table S1). Expression of the *Actin* gene by primer set PchActin131F/PchActin131R (Table S1) was used as an internal control to normalize the data. The primer set NbPDS171F/NbPDS171R was designed for qPCR to detect the expression of *PDS* in *N. benthamiana*, and the expression of the *Actin* gene determined by the primer set NbActin145F/NbActin145R was referred as an internal control (Table S1).

Northern blot assay was employed to detect the expression of the *GFP* gene in *gfp*‐transgenic *N. benthamiana* plants inoculated with PNRSV‐NbGFP100(+) or wtPNRSV at the times indicated. Briefly, total RNA was extracted from leaf tissues using TRIzol (Invitrogen) and separated in 1% agarose gels containing 2% formaldehyde before being transferred to Hybond‐N^+^ membranes (GE Healthcare, Mississauga, ON, Canada). The membranes were incubated with the DIG‐labelled DNA probe, which was prepared as follows. A 150‐bp fragment of GFP was obtained by PCR with primers mGFP(5)150F/mGFP(5)150R (Table S1). The fragment was used to generate a DIG‐dUTP labelled probe using DIG DNA Labelling and Detection Kit (Roche, Mississauga, ON, Canada). Detection of DIG signals was performed according to the manufacturer's instructions (Roche). The total RNA on the agarose gel was stained with ethidium bromide as a loading control.

## Supporting information


**Figure S1** High infectivity of the modified PNRSV infectious clone in *C. sativus*.
**Figure S2** The nt sequence of the *PDS* gene of *N. benthamiana*.
**Figure S3** The nt sequence alignment of the *eIF4E* and *eIF(iso)4E* genes of peach.

**Table S1** Sequences of the primers used in this study.
