# Supplementary material for: An efficient viral vector for functional genomic studies of Prunus fruit trees and its induced resistance to Plum pox virus via silencing of a host factor gene
Source: Plant Biotechnol J. 2016 Sep 29;15(3):344–56. doi: 10.1111/pbi.12629 (PMC5316922; doi:10.1111/pbi.12629)
Supplement: Supplementary file 1 — Figure S1 High infectivity of the modified PNRSV infectious clone in C. sativus. Figure S2 The nt sequence of the PDS gene of N. benthamiana. Figure S3 The nt sequence alignment of the eIF4E and eIF(iso)4E genes of peach. [file PBI-15-344-s001.pdf]

Supporting figures:

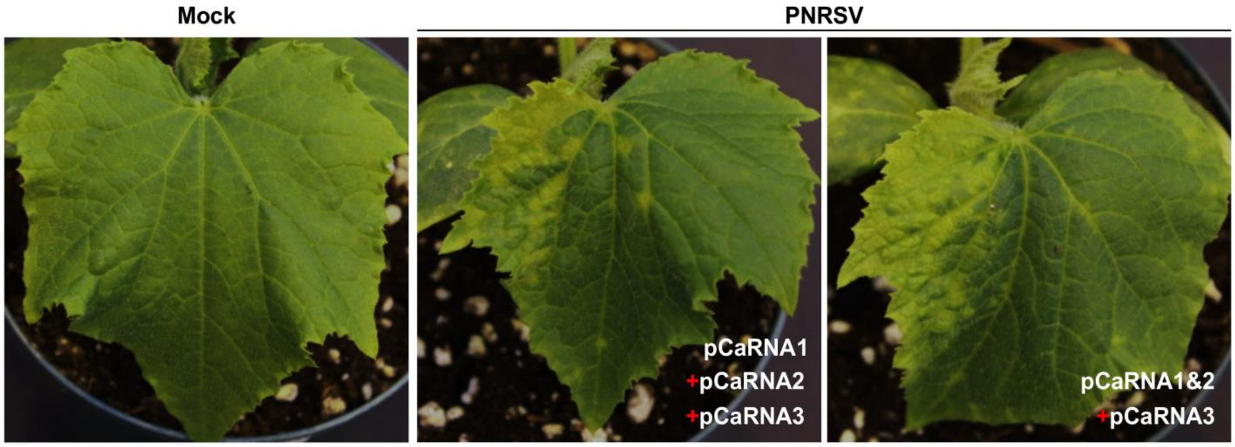

Figure S1. High infectivity of the modified PNRSV infectious clone in *C. sativus*. At 11 dpai, cucumber 'Straight Eight' seedlings inoculated with the modified T-DNA constructs of PNRSV displayed severe ringspot and necrosis symptoms, which were similar with those in plants inoculated with the original PNRSV constructs.

1 ATGCCCCAAATCGGACTTGTATCTGCTGTTAATTTGAGAGTCCAAGGTAATTCAGCTTATCTTTGGAGCTCGAGGTCTTCGTTGGGAACTGAAAGTCAAG

101 ATGTTTGCTTGCAAAGGAATTTGTTATGTTTTGGTAGTAGCGACTCCATGGGGCATAAGTTAAGGATTCGTACTCCAAGTGCCACGACCCGAAGATTGAC

201 AAAGGACTTTAATCCTTTAAAGGTAGTCTGCATTGATTATCCAAGACCAGAGCTAGACAATACAGTTAACTATTTGGAGGCGGCGTTATTATCATCATCG

301 TTTCGTACTTCCTCACGCCCAACTAAACCATTGGAGATTGTTATTGCTGGTGCAGGTTTGGGTGGTTTGTCTACAGCAAAATATCTGGCAGATGCTGGTC

401 ACAAACCGATATTGCTGGAGGCAAGAGATGTCCTAGGTGGGAAGGTAGCTGCATGGAAAGATGATGATGGAGATTGGTACGAGACTGGGTTGCACATATT

501 CTTTGGGGCTTACCCAAATATGCAGAACCTGTTTGGAGAACTAGGGATTGATGATCGGTTGCAGTGGAAGGAACATTCAATGATATTTGCGATGCCTAAC

601 AAGCCAGGGGAGTTCAGCCGCTTTGATTTTCCTGAAGCTCTTCCTGCGCCATTAAATGGAATTTTGGCCATACTAAAGAACAACGAAATGCTTACGTGGC

701 CCGAGAAAGTCAAATTTGCTATTGGACTCTTGCCAGCAATGCTTGGAGGGCAATCTTATGTTGAAGCTCAAGACGGTTTAAGTGTTAAGGACTGGATGAG

801 AAAGCAAGGTGTGCCTGATAGGGTGACAGATGAGGTGTTCATTGCCATGTCAAAGGCACTTAACTTCATAAACCCTGACGAGCTTTCGATGCAGTGCATT

901 TTGATTGCTTTGAACAGATTTCTTCAGGAGAAACATGGTTCAAAAATGGCCTTTTTAGATGGTAACCCTCCTGAGAGACTTTGCATGCCGATTGTGGAAC

1001 ATATTGAGTCAAAAGGTGGCCAAGTCAGACTAAACTCACGAATAAAAAAGATCGAGCTGAATGAGGATGGAAGTGTCAAATGTTTTATACTGAATAATGG

1101 CAGTACAATTAAAGGAGATGCTTTTGTGTTTGCCACTCCAGTGGATATCTTGAAGCTTCTTTTGCCTGAAGACTGGAAAGAGATCCCATATTTCCAAAAG

1201 TTGGAGAAGCTAGTGGGAGTTCCTGTGATAAATGTCCATATATGGTTTGACAGAAAACTGAAGAACACATCTGATAATCTGCTCTTCAGCAGAAGCCCGT

NbPDS300-F

1301 TGCTCAGTGTGTACGCTGACATGTCTGTTACATGTAAGGAATATTACAACCCCAATCAGTCTATGTTGGAATTGGTATTTGCACCCGCAGAAGAGTGGAT

NbPDS200-F

1401 AAATCGTAGTGACTCAGAAATTATTGATGCTACAATGAAGGAACTAGCGAAGCTTTTCCCTGATGAAATTTCGGCAGATCAGAGCAAAGCAAAAATATTG

NbPDS128-F

1501 AAGTATCATGTTGTCAAAACCCCAAGGTCTGTTTATAAAACTGTGCCAGGTTGTGAACCCTGTCGGCCCTTGCAAAGATCCCCTATAGAGGGTTTTTATT

NbPDS-R

1601 TAGCTGGTGACTACACGAAACAGAAGTACATTGGCTTCATGGAAGGTGCTGTCTTATCAGGAAAGCTTTGTGCACAAGCTATTGTACAGGATTACGAGTT

1701 ACTTCTTGGCCGGAGCCAGAAGATGTTGGCAGAAGCAAGCGTAGTTAGCATAGTGAACTAA

Figure S2. The nt sequence of the *PDS* gene of *N. benthamiana*. The nt sequence of *PDS* of *N. benthamiana* was retrieved from GenBank database with the accession number DQ469932. The locations and sequences for the primers NbPDS128-F, NbPDS200-F, NbPDS300-F and NbPDS-R were shown in red letters.

```
eIF4E
1          ATGGTGGTCGAAGACGCACCCAAGACTTCAGCTTCAGAAGATCAGGCCAAGACCGAGACGAACCCTAAACCAAGGGAAGAGGACGATGAGCCCGAAGAAG
1          ATGG----------------------------------CGACAGAGGTAGCAGCAGCAGCAGTTCCG--CCACCACAATTAGACGCTGAG----GAAAAT
eIF(iso)4E ****                                   **    *  *  * *   *  *   *    ***    **   **** ****     * **

101        GAGAGATCGTGGGCGACGAAGAATCGGCGTCGAAGCCATCGAAAGGAATAGCGCCACAGTCTCATGCTCTGGAGCACTCATGGACCTTCTGGTTCGATAG
61         AGTGGGTTAGAGGCGGCGGCGG--CCGAGGCAAAGATAC--AGCCGAGCAGTG----GGCCCCACAAGCTGGAGAGGAAGTGGACCTTTTGGTTCGATAA
               * *    **** **  *   * * * * ***  *   *   **  ** *    * *  **    ******      ******** **********

201        CCCCGCAGCAAAGTCCGCTAAGACTAAGCAAGAGGATTGGGGGAGTTCCATTCGCCCGATCTATACCTTCAGTACCGTCGAGGAGTTCTGGAGCATTTAC
153        CCA---ATCCAAGCCC----AAGCAAGGT--GCTGCTTGGGGTTCCTCTCTTCGCAAGGCCTACACCTTCGAAACCGTTCAAGAATTCTGGTGTCTGTAT
           **    * * *** **    *  * * *   *  * ******    **  *****  *  *** ******   *****  * ** ****** *  * **

301        AATAATATACGTCATCCAAGCAAGTTGGCTCTTGGCACGGACTTCCATTGTTTCAAATATAAAATTGAGCCAAAGTGGGAGGATCCTGTGTGTGCTAATG
244        GATCAGGTATTCAAGCCAAGCAAGTTTCCACCAAATGCAGATTTCCACTTGTTCAGGGCTGGGGTTGAACCAAAATGGGAGGATCCTGAGTGTGCTAATG
            ** *  **    * ***********  * *      * ** ***** *  ****    *    **** ***** ************* ***********

401        GAGGGAAGTGGACTGTAACTTTTCC---TAAAGGGAAATCTGATACTTCTTGGCTGTATACGTTGCTAGGAATGATAGGAGAACAGTTTGATCATGGAGA
344        GAGGGAAGTGGACTGTCACCAGCAGGAGTAAGGCCAGCCTTGATACCATGTGGCTAGAGACCTTGATGGCGCTGATTGGAGAGCAATTTGATGAGGCTGA
           **************** **         *** *  *    ******    *****  * ** *** * *   **** ***** ** ****** * *  **

498        TGAAATTTGTGGAGCAGTTGTCAACGTCAGAAATAGGCAAGAAAAGATATCTATTTGGACGAAGAATGCAATAAATGAAGCTGCTCAGCTGAGCATTGGG
444        TGAGATTTGCGGGGTGGTAGCAAGTGTGCGCCAGAGGCAGGACAAACTTGCATTATGGACTAGGAATGCTGCAAACGAGGCTGCCCAGATGGGCATTGGG
           *** ***** ** *  ** *  *  **  *  * ***** ** **  *  *  * ***** * ******   *** ** ***** *** ** ********

598        AAACAGTGGAAGGGGTTTCTGGATTACAATGAAACTATAGGGTTCATATTTCATGAGGATGCAATGAGACAAGAGAGAAGTGCCAAAAATAAATACGTAG
544        AGGAAGTGGAAGGAGATAATCGATGTCACTGATAAGATCACCTACAGCTTCCATGACGATTCTAAAAGG---GAAAGATCAGCAAAGCCTCGATACAATG
           *   ********* * *  * ***  ** *** *  **    * **  ** ***** *** * *  **    ** ***   ** **   *  ****   *

698        CCTGA
641        TTTGA
             ***
```

Figure S3. The nt sequence alignment of the *eIF4E* and *eIF(iso)4E* genes of peach. Both *eIF4E* and *eIF(iso)4E* sequences were retrieved from the PDR database (http://www.rosaceae.org/species/prunus_persica/genome_v1.0) with accession numbers ppa010850m and ppa011357m, respectively. The 120-bp sequence of the *eIF(iso)4E* gene (in red) was used to construct pCaRNA3-eIFiso4E120(+).
