# Supplementary material for: An efficient viral vector for functional genomic studies of Prunus fruit trees and its induced resistance to Plum pox virus via silencing of a host factor gene
Source: Plant Biotechnol J. 2016 Sep 29;15(3):344–56. doi: 10.1111/pbi.12629 (PMC5316922; doi:10.1111/pbi.12629)
Supplement: Supplementary file 2 — Table S1 Sequences of the primers used in this study. [file PBI-15-344-s002.docx]

| Name | Sequence | Description |
| --- | --- | --- |
| HindⅢ-35S-F | CGATTTCTGAAGCTTGCATGCCT | amplify complete RNA1 expression cassette of PNRSV from pCaRNA1 |
| HindⅢ-35ST-R | ATATAAAGCTTAGGCGATTAAGTTGGGTAAC |  |
| RNA3-3’UTR-R | GCTTCCCTAACGGGGCATCC | PCR identification of copy number and sense/antisense orientation of foreign insert in pCaRNA3 backbone |
| RNA3-CP-F | AGGTCTTGGTTAGGGATTTG |  |
| NbPDS128-F | TACATTCTAGATTGTCAAAACCCCAAGGTC | Designed according to the sequence DQ469932 (GenBank); paired with the primer NbPDS-R to amplify a 128-, 200- or 300-bp fragment of *N. benthamiana* PDS gene; to construct pCaRNA3-PDS128(+), pCaRNA3-PDS128(−), pCaRNA3-PDS200(+), pCaRNA3-PDS200(−), pCaRNA3-PDS300(+) and pCaRNA3-PDS300(−). |
| NbPDS200-F | TACATTCTAGAAGGAACTAGCGAAGCTTT |  |
| NbPDS300-F | TACATTCTAGAGAATATTACAACCCCAAT |  |
| NbPDS-R | TACATTCTAGATGAAGCCAAGTACTTCTG | Designed according to the sequence DQ469932 (GenBank) |
| mGFP(5)100(+) | TACATCCTAGGATGAGTAAAGGAGAAGAACT | Designed according to the sequence U87973 (GenBank); to amplify a 100-bp GFP fragment of *gfp*-transgenic *N. benthamiana*; to construct pCaRNA3-GFP100(+) |
| mGFP(5)100(-) | TACATCCTAGGCACCCTCTCCACTGACAGAA |  |
| PchPDS100(+) | TACATCCTAGGAGAAAGCTGAAGAACACATA | Designed according to the sequence AY822065 (GenBank); to amplify a 100-bp fragment of peach PDS gene; to construct pCaRNA3-pchPDS100(+) |
| PchPDS100(-) | TACATCCTAGGGATTGTAATATTCCTTACAT |  |
| mGFP(5)150+ | AAGGTGATGCAACATACGGA | Designed according to the sequence U87973 (GenBank); to amplify a 150-bp GFP fragment of *gfp*-transgenic *N. benthamiana* (16c);  northern blot detection of GFP expression |
| mGFP(5)150- | AGAAGTCGTGCCGCTTCATA |  |
| NbPDS171F | TGGGAAGGTAGCTGCATGGAAAGA | Designed according to the sequence DQ469932 (GenBank); real-time PCR detection of PDS gene expression of *N. benthamiana* |
| NbPDS171R | CCTGGCTTGTTAGGCATCGCAAAT |  |
| NbActin145F | AAAGACCAGCTCATCCGTGGAGAA | Designed according to the sequence AY179605 (GenBank); real-time PCR detection of internal reference Actin gene of *N. benthamiana* |
| NbActin145R | TGTGGTTTCATGAATGCCAGCAGC |  |
| pchPDS145F | CCTTGCAAAGATCTCCCCTA | Designed according to the sequence AY822065 (GenBank); real-time PCR detection of PDS gene expression of peach |
| pchPDS145R | CGAGCAACAAGCAATTCGTA |  |
| PchActin131F | TACGAAGGTTATGCGCTTCC | Designed according to the sequence ppa007238m (PDR, <https://www.rosaceae.org/species/prunus_persica/genome_v1.0>); real-time PCR detection of internal reference Actin gene of peach |
| PchActin131R | ACAATTTCCCGTTCAGCAGT |  |
| pch(iso)1F | TACATTCTAGAATGGCGACAGAGGTAGCAGCAGCAG | Designed according to the sequence ppa011357m (PDR, <https://www.rosaceae.org/species/prunus_persica/genome_v1.0>); to amplify a 120-bp fragment of peach eIF(*iso*)4E gene;  to construct pCaRNA3-eIFiso4E120(+) |
| pch(iso)1R | TACATTCTAGACTTGTGGGGCCCACTGCTCGGCTGT |  |
| q4E-F | AAAATTGAGCCAAAGTGGGA | Designed according to the sequence ppa010850m (PDR, <https://www.rosaceae.org/species/prunus_persica/genome_v1.0>); real-time PCR detection of eIF4E gene expression of peach |
| q4E-R | TTCTCCTATCATTCCTAGCA |  |
| qiso4E-F | CAGGACAAACTTGCATTATGGA | Designed according to the sequence ppa011357m (PDR, <https://www.rosaceae.org/species/prunus_persica/genome_v1.0>); real-time PCR detection of eIF(*iso*)4E gene expression of peach |
| qiso4E-R | CATGGAAGCTGTAGGTGATCT |  |

Table S1. Sequences of the primers used in this study
